# Estimating Neural Background Input with Controlled and Fast Perturbations: A Bandwidth Comparison between Inhibitory Opsins and Neural Circuits

**DOI:** 10.3389/fncir.2016.00058

**Published:** 2016-08-15

**Authors:** David Eriksson

**Affiliations:** ^1^Center for Neuroscience, Albert Ludwig University of FreiburgFreiburg, Germany; ^2^BrainLinks-BrainTools, Albert Ludwig University of FreiburgFreiburg, Germany

**Keywords:** perturbation, optogenetic inhibition, side effects, controlled perturbation, contextual input

## Abstract

To test the importance of a certain cell type or brain area it is common to make a “lack of function” experiment in which the neuronal population of interest is inhibited. Here we review physiological and methodological constraints for making controlled perturbations using the corticothalamic circuit as an example. The brain with its many types of cells and rich interconnectivity offers many paths through which a perturbation can spread within a short time. To understand the side effects of the perturbation one should record from those paths. We find that ephaptic effects, gap-junctions, and fast chemical synapses are so fast that they can react to the perturbation during the few milliseconds it takes for an opsin to change the membrane potential. The slow chemical synapses, astrocytes, extracellular ions and vascular signals, will continue to give their physiological input for around 20 ms before they also react to the perturbation. Although we show that some pathways can react within milliseconds the strength/speed reported in this review should be seen as an upper bound since we have omitted how polysynaptic signals are attenuated. Thus the number of additional recordings that has to be made to control for the perturbation side effects is expected to be fewer than proposed here. To summarize, the reviewed literature not only suggests that it is possible to make controlled “lack of function” experiments, but, it also suggests that such a “lack of function” experiment can be used to measure the context of local neural computations.

## Introduction

**Figure 1:**
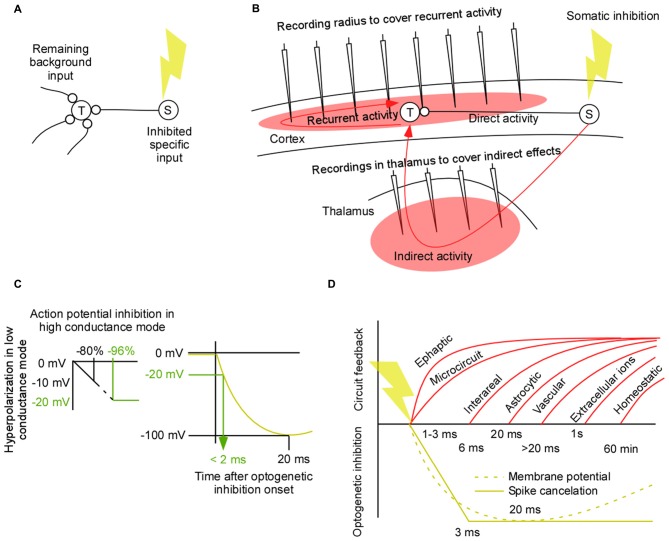
**Controlled perturbations. (A)** A lack of function experiment in which the source population (S) is inhibited and the modified activity in the target population (T) is recorded. Such an experiment may be used to estimate the context of a neural computation in terms of the background signal. **(B)** A worst case scenario for a “lack of function” experiment and for an estimation of the background/context signal of local computations in a corticothalamic circuit. The slow and variable feedback axons from the perturbed area to the target area makes it difficult to isolate the direct effect from the perturbed area (S) and the indirect and recurrent effects to area (T). To understand those effects it is necessary to record them. This review addresses which areas we need to record from to cover the side effects of a perturbation. **(C)** Estimation of the time it takes to inhibit 96% of the spikes in a high conductance state. A linear approximation of the relation between hyperpolarization in low conductance mode and action potential inhibition in high conductance mode (left). Illustration of the non-linear relation between time and hyperpolarization for inhibitory opsins (right). **(D)** Neural signal types and their feedback latencies. Ephaptic latencies are less than 3 ms (Yim et al., 1986). Gap junction latencies are less than 2.5 ms (Long et al., 2002; Hu and Bloomfield, 2003; Bennett and Zukin, 2004). Neuron to neuron short range chemical synapse latency is 1–2 ms (Feldmeyer et al., 2006; Boudkkazi et al., 2007). Neuron to neuron long range feedback latency from cortex to thalamus is 2–36 ms (Briggs and Usrey, 2009). Noradrenergic pathways delays are 30–132 ms (Aston-Jones et al., 1985). Dendritic propagation can add up to 6 ms when the synapse is at distal dendrites (Sjöström and Häusser, 2006). Neuron to neuron axonal conduction delays add around 0.3 mm/ms for intracortical connections (Luhmann et al., 1990; Hirsch and Gilbert, 1991; Murakoshi et al., 1993; Bringuier et al., 1999; Nauhaus et al., 2009). Neuro to glia latency = 20 ms (Lind et al., 2013). Within glia membrane potential changes are slower than 20 ms (Mishima and Hirase, 2010). Glia to neuron latency is estimated to be less than 20 ms (Sasaki et al., 2012). Glia to glia via calcium induced signaling may, in general, be an order of magnitude slower (Amzica and Steriade, 2000; Amzica and Massimini, 2002; Nadkarni and Jung, 2004). Glia to vasculature signaling latencies are longer than 0 ms (Lind et al., 2013). This in turn gives a neuron to vasculature signaling that is slower than the neuron to glia signaling. Ionic changes in the extracellular medium can evoke changes in the membrane potential with a time constant of seconds to minutes (Ferenczi et al., 2016). Homeostatic changes typically takes minutes to hours (Turrigiano et al., 1998; Kim et al., 2011; Mitra et al., 2012).

Many times in neuroscience it is necessary to prove the importance of one type of population of neurons. Since the “neuronal code” is unknown it is difficult to test the function by stimulation or depolarization. Instead a better option is to inhibit the population to test the “lack of function” (Zhang et al., 2007). As a result of inhibiting the source population (S) the activity in a downstream target population (T) will be modified (Figure 1A). This remaining activity resembles the background activity that is coming from all over the brain except from the inhibited area. Since this remaining background activity defines the context of neural computations we propose that it should be estimated. Furthermore, if we manage to estimate this activity we have also found a way to make “lack of function” experiments more controlled.

As we will show in this article the most demanding circuits to perturb is that of modulatory feedback pathways (Figure 1B). If we have the means to perturb feedback pathways in a controlled manner we are in a good position to perturb any circuit in the brain. The quick spread through the densely connected network can make any perturbation unspecific even after a few tens of milliseconds. Therefore it would be ideal to limit this spread by means of short perturbations (El-Boustani and Sur, 2014; El-Boustani et al., 2014). By minimizing the spread we have the chance to record the side effects of the perturbation (red filled areas in Figure 1B). Two main classes of pathways contribute to those side-effects; recurrent and indirect pathways (red lines in Figure 1B). As the wave of perturbed physiological activity starts from population (S) it will not only propagate to population (T) through the direct path; it will also propagate to population (T) via indirect areas. Once this direct/indirect wave has reached population (T) it will trigger recurrent pathways. Thus recurrent side-effects have their origin in population (T) and indirect side-effects have their origin in population (S). Both influence population (T). Before we review the latency of those side-effects we first examine the speed of optogenetic inhibition.

## Fast Optogenetic Inhibition

Ultimately we would like to inhibit spikes rapidly. Before we turn to spike inhibition we briefly review the results of optogenetic membrane potential hyperpolarization using opsins. The rise time of the hyperpolarizing current for different hyperpolarizing opsins is in the range of 2 and 3 ms (Mattis et al., 2012). Using the halorhodopsin, eNpHR3.0, the membrane potential in a cell culture can be hyperpolarized with 100 mV within approximately 20 ms (Gradinaru et al., 2010). How strong hyperpolarization is required to cancel spikes *in vivo* in an awake animal is difficult to estimate due to the high conductance/up-state when the neural circuit is highly active (Destexhe et al., 2003). Although the injection of a negative current of −0.62 nA generates a strong hyperpolarization of the down-state at resting conditions, approximately −12 mV, it generates a much weaker hyperpolarization of the up-state, approximately −2 mV (Paré et al., 1998). On the other hand, the same current injection results in an elimination of roughly 80% of the spikes in Figure 4A in Paré et al. (1998). Since the membrane potential hyperpolarizations typically are made in the cell culture and since the silencing of spikes will be done in the high conductance state we have related those two (Figure 1C). Given this example, a strong firing rate decrease of 96% is expected for a −20 mV hyperpolarization. To estimate how fast a −20 mV hyperpolarization can be achieved with eNpHR3.0 we have combined the hyperpolarization amplitude of Figure 1E in the article of Gradinaru et al. (2010) with the voltage trace of the NpHR1.0 in Figure 1D in Zhang et al. (2007; Figure 1C). By shrinking the curve from Zhang et al. (2007) in time, such that the peak comes at 20 ms, the latency of a −20 mV hyperpolarization is estimated to be less than 2 ms.

This quick inhibition is also supported by the fact that action potentials can be silenced using halorhodopsin *in vivo* in the awake behaving mouse within 3 ms, as indicated by a near complete drop in the population spike count from one 30 ms bin to the next (English et al., 2012). By quantifying the drop in spike counts, we calculated a meta-analytic inhibition delay of 3 ± 2.1 ms (English et al., 2012; Courtin et al., 2014). In contrast to this quick inhibition it was shown that neurons in the behaving macaque monkey were inhibited with an average time constant of 60 ms (Han et al., 2011). This long inhibition time is most likely because all neurons were pooled whether they expressed the inhibitory opsin or not. Therefore some of those neurons will be influenced indirectly via differentially delayed signal paths.

How do one optimize the conditions for achieving fast somatic inhibition? A strong expression level is essential for evoking fast inhibition. Although the expression level depends on many factors such as promotor, virus, titer, expression duration, it suffices to say that expression rates of up to 80% have been reported for adeno associated viruses (Diester et al., 2011; Klein et al., 2016). In order to be able to inhibit the soma of cells that are projecting to a specific area it is possible to induce retrograde synaptic transport using the pseudo rabies virus (Wickersham et al., 2007a,b) or using the canine adenovirus (CAV) virus (Hnasko et al., 2006). With the pseudo rabies virus around 10% of the presynaptic neurons are assumed to be infected (Callaway and Luo, 2015). A less toxic version of the rabies virus strain has recently been shown to infect an order of magnitude more presynaptic neurons thereby providing close to optimal conditions for a projection specific “lack of function” experiment (Reardon et al., 2016). High illumination intensity is also expected to decrease the latency (Braun and Hegemann, 1999). Although a high illumination intensity might not have been possible in several of the previous studies using illumination lasting seconds to minutes (Chow et al., 2010; Han et al., 2011; McCutcheon et al., 2014; Spellman et al., 2015), the light intensity can be higher for a short lasting inhibition (Stujenske et al., 2015). In order to inhibit neurons, the light should be directed towards proximal cell compartments, preferably somata, since the large electronic distances attenuates and slows down the dendritic voltage changes (Liu et al., 2012, 2014; Ramirez et al., 2013; Cowansage et al., 2014; Redondo et al., 2014). The somata can either be targeted selectively with small volume, low intensity stimulation (Zorzos et al., 2010; Hayashi et al., 2012; Kruse et al., 2014; Pisanello et al., 2014), or non-selectively with large volume, high intensity, and pulsed stimulation (Stujenske et al., 2015).

A powerful way to inhibit neurons is to excite inhibitory neurons. Channelrhodopsin can be targeted specifically to parvalbumin expressing inhibitory neurons by means of a transgenic mouse line. Since multiple parvalbumin interneurons target proximal neurites and somata of each pyramidal cell, the inhibition can be both fast and strong. Using a relatively low illumination intensity, the firing of pyramidal cells reaches maximal inhibition within 3 ms (Cardin et al., 2009; Siegle et al., 2014), and with a light power density of approximately 10 mW/mm^2^, 90% of the cells could be silenced (Li et al., 2016).

The output of neurons can also be inhibited by inhibiting their axons or their terminals directly (Tye et al., 2011; Spellman et al., 2015; Mahn et al., 2016). In a recent study it has been shown that long duration axonal inhibition (seconds to minutes) can lead to excitatory effects for certain opsins (Mahn et al., 2016). This may, however, be a smaller problem for the short inhibition durations that we propose. To the best of the author’s knowledge there is, however, no study that systematically estimates the time it takes to inhibit axonal terminals *in vivo*.

To summarize, the reviewed literature suggests that somatic optogenetic inhibition cancels spikes within 3 ms. Expression efficiencies of 80% has been reported in the literature indicating close to optimal conditions for “lack of function” experiments. Finally, it is not unlikely that one can achieve a near 100% inhibition by driving inhibitory neurons. Also this inhibition is expected to take 3 ms. Now we will compare this inhibition latency to the feedback latency of neuronal circuits.

## Recurrent Effects

As the perturbation wave reaches population (T) it will respond via many recurrent mechanisms. Slow chemical synapses, astrocytes, vasculature, extracellular ions will react with a latency of more than 20 ms (Figure 1D). Importantly, after the perturbation onset those slow signal-types will continue to give their natural input during around 20 ms. Thus if we record the membrane potential and/or firing pattern in the target neurons we can estimate this slow component of the background signal. The recorded activity will also contain a faster component from ephaptic effects, gap-junctions, and many types of chemical synapses. The difference to the slow component is that this input will be a modified version of the fast natural input since those fast effects react during the onset of the perturbation within 2–3 ms. Thus we need to do additional recordings from the neurons that contribute to this fast effect if we want to understand how they influence the fast component.

Slower recurrent effects come from intra and inter-areal connections. The latency of recurrent, intra-areal connections add around 3 ms/mm (Luhmann et al., 1990; Hirsch and Gilbert, 1991; Murakoshi et al., 1993; Bringuier et al., 1999; Nauhaus et al., 2009). A recurrent reaction to a perturbation is therefore expected after more than 2 * 3 = 6 ms/mm. Functional estimates of recurrent inter-areal latencies suggest latencies longer than 6 ms (Bastos et al., 2015). The most frequent axons from cortex to thalamus have a delay of 5 ms (Ferster and Lindström, 1983), and the axons back to cortex have a delay of around 1 ms (Cleland et al., 1976).

In general, recurrent effects will be a combination of intra- and inter areal connections spanning multiple areas. Due to the small world network architecture of the brain, a perturbation can reach all cells in the brain within a relatively short time. The short distance between nodes in a small world network is because there are some random connections that connect distant areas. As a result, only a few synapses may connect any pair of neurons in the brain (Sporns et al., 2004; van den Heuvel and Sporns, 2011), and up to a theoretical estimate of ln (100,000,000) = 18 synapses in the mouse with 100,000,000 neurons (Watts and Strogatz, 1998). The average wiring length between two neurons has been estimated to 35 mm in the macaque monkey (Kaiser and Hilgetag, 2006), and if we assume a relatively slow conduction velocity of 0.3 mm/ms (see above) we end up with a communication time of around 35 mm/0.3 mm/ms = 100 ms. Although this suggests that a perturbation has the potential to spread quickly in the brain, the next question is how the perturbation decays as it spreads. In one elegant study it was shown that the propagation of an electrical perturbation diminishes already after one synapse. The limited propagation may be due to the recruitment of a strong inhibition since the electrical stimulation activates many neurons synchronously (Logothetis et al., 2010). If the perturbation is more biologically accurate, or inhibitory, it is possible that the perturbation will reach further synapses. Thus if the inhibition is longer than 100 ms we may need to record from all areas that are connected to population (T) in order to understand how the perturbation affects it. In addition, we would need to record astrocytic and vascular signals. An additional complication is represented by the compensations governed by the large variety of homeostatic, reverberating and plasticity related effects. For this reason, and for our purposes, it does not make sense to make chronic lesions (Otchy et al., 2015). On the contrary, when we make a short lasting perturbation we have the chance to record and control for the recurrent activity.

## Indirect Effects

**Figure 2:**
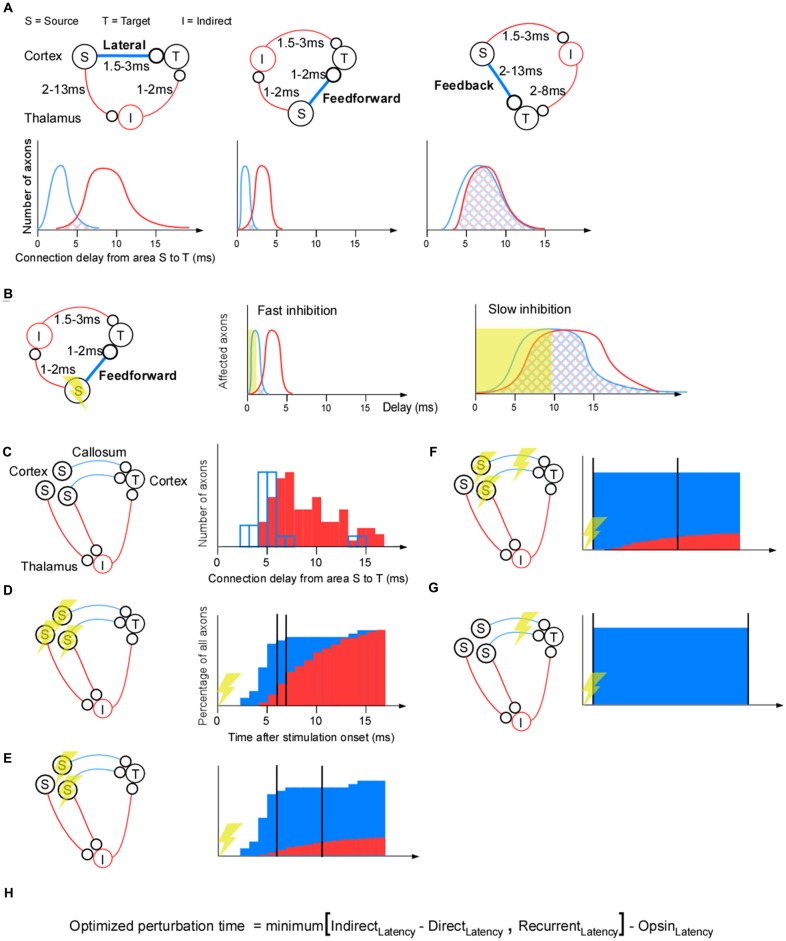
**Separating indirect effects from direct effects for a corticothalamocortical pathway. (A)** The lateral corticocortical projection (left), feedforward thalamocortical projection (middle), and feedback corticothalamic projection (right). Cortex is represented by the upper half of the panels, thalamus by the lower half (so S and T, I and T, or S and I lie in cortex in the three subpanels, respectively). The conduction delays for the feedforward, lateral, and feedback cases are estimates from the literature (Miller, 1975; Cleland et al., 1976; Ferster and Lindström, 1983; Feldmeyer et al., 2006; Boudkkazi et al., 2007). Below each connectivity diagram is a schematic illustration of the delay distribution of the axons along the direct (blue) and indirect (red) pathways. For example, for the indirect pathway, a slow S-I axon and a fast I-T axon will be counted as the same axonal delay as a fast S-I axon and a slow I-T axon. If those two examples sums up to the same delay they will contribute with two “axons” to the “number of axons” for that delay. **(B)** The time it takes to achieve complete inhibition dictates the degree of overlap between the direct and indirect pathways. **(C–G)** Four different ways to apply optogenetic inhibition. **(C)** As a quantative example, we have chosen a corticocortical connection as the direct connection (blue lines), and a corticothalamocortical pathway as the indirect (red lines) connection. The corticocortical connection delays are taken from somatosensory cortico-cortical axons across callosum that were electrically stimulated and revealed by collision (modified with permission; Miller, 1975). Delays in the indirect pathway were estimated by adding 2 ms to the corticothalamic conduction (modified with permission; Ferster and Lindström, 1983) delay in order to take into account for the axon to postsynaptic potential (PSP) delay (Ferster and Lindström, 1983), the conversion time from PSP to an action potential delay, and the thalamocortical conduction delay (Cleland et al., 1976). **(D)** Projection-unspecific somatic inhibition. The cumulative histogram has been calculated from the delay histograms in **(C)** to quantify the percentage of indirect and direct axons that have been active up to that point in time. The indirect path can be separated from the direct path if, for example, more than 80% of the direct connections have had an effect, and less than 30% of the indirect connections have had an effect (two vertical black lines). **(E)** Projection-specific somatic inhibition. The cumulative histogram of the indirect path has been shrunk relative to the one in **(D)** to illustrate that fewer indirect pathways are affected by the perturbation if it is selective for direct pathways. **(F)** Axonal inhibition and somatic inhibition. The cumulative histogram for direct axonal inhibition is an illustration showing that all axons may be inhibited simultaneously and early since axonal inhibition eliminates the axonal propagation time. **(G)** Axonal inhibition only. **(H)** Formula for calculating an optimal perturbation duration. Recurrent_Latency_ = latency of the fastest non-recorded recurrent path. If we record from a recurrent path with a short latency this path should not limit the perturbation time, since through that recording, we know the effects of that recurrent path. Thus the more additional recordings we make in short latency paths the longer the perturbation time can be. Indirect_Latency_ = latency of the fastest non-recorded indirect path. This will be infinite, ∞, for axonal inhibition, which in turn makes the recurrent latency the limiting term. Direct_Latency_ = Latency of direct path (~1 ms for axonal inhibition). Opsin_Latency_ = Ramping-up time of opsin (assume 3 ms). Note that this formula does not take into account axonal latency variability: the variability is assumed to be 0.

To recapitulate, optogenetic inhibition is faster than the recurrent feedback from glia, vasculature, extracellular ions and slow chemical synapses. Next we ask if indirect polysynaptic paths between population (S) and population (T) may be slower than the direct path between those populations. This would be advantageous since fewer “side-effect” recordings of indirect paths would be necessary. For any indirect path there will per definition be at least one more synapse than for the direct pathway. Thus indirect pathways should on average be slower than direct pathways. Therefore the two types of pathways may separate in time. A complication, however, is that the heterogeneity in axonal conduction velocities will cause temporal dispersion, or broadening of the wavefront, along the direct and indirect paths, and hence lead to an increased temporal overlap between them. Thus if one have the choice one should study a pathway for which the indirect pathways are weaker and slower than the direct pathway. In general, driving, feedforward connections have exactly this feature of being strong, fast and showing little temporal variability (Reichova and Sherman, 2004). This is in sharp contrast to modulating feedback pathways that may be weaker and slower. Therefore, in general, the overlap between the direct and indirect pathways will be largest for the feedback pathways (Figure 2A).

A proper choice of the type of optogenetic inhibition can decrease the overlap between the direct and the indirect pathways. First, to minimize the overlap, one should inhibit for as short a duration as possible (Figure 2B). Second it matters which cells one stimulates and if one applies somatic or axonal inhibition. This is illustrated in the corticothalamocortical example where the indirect pathway goes through the thalamus (Figure 2C).

If we apply nonspecific somatic inhibition or excitation of inhibitory neurons the amplitude of the indirect signal will be maximal (Figure 2D). This is because all neurons that project to indirect areas will be inhibited, in addition to those needed for the direct projection. For example, after 6 ms inhibition, most of the direct axons are influenced and a smaller proportion of the indirect axons are influenced (the left vertical black line in Figure 2D). From this time point we can record the activity for at most 1 ms (the right vertical black line in Figure 2D) in population (T) before a significant part of the indirect pathway is affected. For longer inhibition durations we would need to record from the indirect neurons (I) in order to understand their effect. To reduce inhibition of the indirect signal, only directly projecting neurons should be inhibited. This increases the separation between the direct and indirect pathway (Figure 2E).

Direct and indirect signals can be separated in time if axons that project to population (T) are inhibited (Figure 2F). Since the presynaptic terminals of the direct path will be synchronously inhibited, the temporal delay and spread (owing to heterogeneous axonal conduction velocity) will be eliminated. Thus, the temporal perturbation jitter will now be reduced to the variability in synaptic latency (i.e., around 1 ms; Boudkkazi et al., 2007). Finally, to avoid indirect effects, the best inhibition strategy would be to only inhibit the axons, and not the cell bodies (Figure 2G).

So far we have dealt with direct pathways that are monosynaptic. A distinction between direct and indirect pathways is especially difficult when the direct pathway spans multiple synapses, such as from a central brain area to the motor neurons. Therefore, unless one perturb an area close to the motor neurons (e.g., spinal cord), the interpretation of behaviors may be non-trivial (Otchy et al., 2015; Sudhof, 2015). Furthermore, the closer population (S) is to the motor neurons the easier it will be to observe an impact on movement output of the short lasting inhibition. Nevertheless it should be noted that even if a central area, far away from the motor neurons, is perturbed with extremely brief epochs of cortical activity in a sparse subset of pyramidal neurons, this is enough to change behavior (Huber et al., 2008). To control for the side effects of a perturbation it may be advisable to study monosynaptic pathways in general, and in particular feedforward/driving pathways using somatic inhibition, and feedback pathways using axonal inhibition. Once those mono-synaptic paths are understood it may be easier to understand the polysynaptic pathways governing behavior.

## Conclusion

A formula for calculating the optimized perturbation time of a “lack of function” experiment or background signal estimation is shown in Figure 2H. If the direct path from (S) to (T) in Figure 1B is a feedback path with a long latency (e.g., 10 ms), it is only possible to achieve a positive optimal perturbation time using both axonal inhibition and additional recordings in the target population (T); axonal inhibition with recordings in a 1 mm radius gives a 3 ms perturbation time: minimum(∞, 2 * 1 mm/0.3 mm/ms) − 3 = 3 ms. In contrast, unrecorded side-effects will result in a negative perturbation time for somatic inhibition (assuming indirect feedback latency of 6 ms): minimum(6–10, 2) − 3 = −7 ms, or for axonal inhibition without additional recordings: minimum(∞, 2) − 3 = −1 ms. To conclude, somatic inhibition is appropriate for feedforward pathways with their small latency/variability and strong connection, whereas axonal inhibition would be necessary for feedback pathways with their large latency/variability and weak connection. By tailoring the optogenetic inhibition to the circuit at hand it may be possible to record all perturbation side effects of some of the more demanding brain circuits.

## Author Contributions

The author conceived and performed the study.

## Funding

The article processing charge was funded by the German Research Foundation (DFG) and the University of Freiburg in the funding programme Open Access Publishing.

## Conflict of Interest Statement

The author declares that the research was conducted in the absence of any commercial or financial relationships that could be construed as a potential conflict of interest.
